# HIV serostatus and tumor differentiation among patients with cervical cancer at Bugando Medical Centre

**DOI:** 10.1186/1756-0500-5-406

**Published:** 2012-08-04

**Authors:** Dismas Matovelo, Moke Magoma, Peter Rambau, Anthony Massinde, Nestory Masalu

**Affiliations:** 1 Department of Obstetrics & Gynecology, Bugando Medical Centre, P.O. Box 1370, Mwanza, Tanzania; 2 Department of Pathology, Catholic University of health and allied sciences, P.O. Box 1464, Mwanza, Tanzania; 3 Department of Oncology, Bugando Medical Centre, P.O. Box 1370, Mwanza, Tanzania

**Keywords:** HIV, Cervical cancer, Clinical stage, Tumor differentiation, Tanzania

## Abstract

**Background:**

Evidence for the association between Human immunodeficiency virus infection and cervical cancer has been contrasting, with some studies reporting increased risk of cervical cancer among HIV positive women while others report no association. Similar evidence from Tanzania is scarce as HIV seroprevalence among cervical cancer patients has not been rigorously evaluated. The purpose of this study was to determine the association between HIV and tumor differentiation among patients with cervical cancer at Bugando Medical Centre and Teaching Hospital in Mwanza, North-Western Tanzania.

**Methods:**

This was a descriptive analytical study involving suspected cervical cancer patients seen at the gynaecology outpatient clinic and in the gynaecological ward from November 2010 to March 2011.

**Results:**

A total of 91 suspected cervical cancer patients were seen during the study period and 74 patients were histologically confirmed with cervical cancer. The mean age of those confirmed of cervical cancer was 50.5 ± 12.5 years. Most patients (39 of the total 74–52.7%) were in early disease stages (stages IA-IIA). HIV infection was diagnosed in 22 (29.7%) patients. On average, HIV positive women with early cervical cancer disease had significantly more CD4+ cells than those with advanced disease (385.8 ± 170.4 95% CI 354.8-516.7 and 266.2 ± 87.5, 95% CI 213.3-319.0 respectively p = 0.042). In a binary logistic regression model, factors associated with HIV seropositivity were ever use of hormonal contraception (OR 5.79 95% CI 1.99-16.83 p = 0.001), aged over 50 years (OR 0.09 95% CI 0.02-0.36 p = 0.001), previous history of STI (OR 3.43 95% CI 1.10-10.80 p = 0.035) and multiple sexual partners OR 5.56 95% CI 1.18-26.25 p = 0.030). Of these factors, only ever use of hormonal contraception was associated with tumor cell differentiation (OR 0.16 95% CI 0.06-0.49 p = 0.001). HIV seropositivity was weakly associated with tumor cell differentiation in an unadjusted analysis (OR 0.21 95% CI 0.04-1.02 p = 0.053), but strong evidence for the association was found after adjusting for ever use of hormonal contraception with approximately six times more likelihood of HIV infection among women with poorly differentiated tumor cells compared to those with moderately and well differentiated cells (OR 5.62 95% CI 1.76-17.94 p = 0.004).

**Conclusion:**

Results from this study setting suggest that HIV is common among cervical cancer patients and that HIV seropositivity may be associated with poor tumour differentiation. Larger studies in this and similar settings with high HIV prevalence and high burden of cervical cancer are required to document this relationship.

## Background

Cancer of the cervix is the second most common cancer among women worldwide with an estimate of 529,409 new cases and 274,883 deaths each year [1-4]. About 80% of cervical cancer cases occur in developing countries. The age-standardized incidence rate in Sub-Saharan Africa is 30–67 per 100,000 which is two to ten times higher than that in developed countries [3]. In Tanzania, cervical cancer is the leading malignancy among women [1]. The incidence rate of the disease in the country is estimated at 40.6 per 100,000 women with approximately 7515 new cases annually [1,5].

The majority of cases are squamous cell carcinoma (85%) and adenocarcinomas (10-12%). Several other types of carcinoma such as adenosquamous carcinoma, adenoid cystic carcinoma, small cell carcinoma and mucinous cancers make up the remaining 3-5% of all cases [1].

The etiology and natural history of cervical cancer is well understood. The proximate causative factor is infection with the high-risk HPV types including 16, 18, 33 & 45. The high-risk HPV types 16 and 18 are the most frequent types and cause about 70% of cancer of cervix worldwide [6,7]. Other proposed independent risk factors for developing cervical cancer include long term use of oral contraceptives, smoking, age at menarche, age at menopause and behaviour which exposes one to have HPV infection such as early sexual debut, increased number of lifetime sexual partners and history of sexually transmitted diseases [6,7].

Cervical cancer commonly affects women of middle or older age, but with era of HIV/AIDS it may be diagnosed in any reproductive age women as HIV infection appears to enhance HPV proliferation [8,9]; although the mechanism by which HIV increases risk of cervical cancer is not clearly understood. It is suggested that HIV induced immunosuppression reduces the ability of the body’s immune system to control the expression of HPV and the production of HPV oncoproteins E6 and E7. The risk appears to be associated with increased HPV persistence and reactivation that may result from immunosuppression related to HIV. The risk is greater in women with low CD4+ counts, especially those with less than 200 cells/μL and in those with high plasma HIV-RNA levels. Further; HIV infection is associated with high risk HPV types known to cause invasive cervical cancer [9-11]. Specifically for Sub-Saharan Africa, more prevalent high-risk and diverse HPV types linked to HIV may exist than elsewhere [8], thus the observed association.

Worldwide, cancer of the cervix has been included as one of the AIDS-defining illnesses and an increased risk of invasive cervical cancer among HIV positive women is documented [4,12,13]. Nevertheless, cervical cancer as a leading malignancy among women not withstanding [1], the magnitude of HIV infection among cervical cancer patients is still not well known in most low-resourced countries burdened with high levels of HIV infection and cervical cancer. In Tanzania, for example, women with cervical cancer are not routinely tested for HIV. In addition, lack of a cancer registry in the country does not allow retrospective analysis for factors associated with various cancers, including HIV. Thus, studies on factors associated with cancers in a clinical setting remain the best opportunity to document this relationship, albeit at limited magnitude. We conducted a descriptive and analytical study to document the association between HIV infection and cervical cancer cell differentiation at Bugando Medical Centre- a tertiary referral and teaching hospital in Mwanza, North-west Tanzania.

## Methods

This was a descriptive analytical study involving suspected cervical cancer patients. The study was carried out in the gynecological outpatient clinic and gynecological ward between November 2010 and March 2011. Bugando Medical Centre serves about 13 million people from six regions of Mwanza, Kagera, Shinyanga, Mara, Kigoma and Tabora in the Lake Zone. The Hospital has a bed capacity of approximately 800. It also has a well equipped pathology laboratory manned by two pathologists. About 3–4 women with suspected cancerous lesion of the cervix are seen weekly at the hospital for investigation and initial care before radiotherapy. Approximately 389 cases of cervical cancer are seen yearly [14].

The study population included all consecutive women with suspicious cervical cancer lesions who were either admitted in gynecological wards or seen at the gynecological outpatient clinic. Women were excluded if they were unconscious without next of kin to consent on their behalf.

Invasive cervical cancer was diagnosed histologically if the cancer had made stromal invasion of at least 3 mm and 7 mm in depth and width respectively. Suspected cervical cancer patients were those with contact vaginal bleeding/postmenopausal bleeding, abnormal vaginal discharge, fecal/urine incontinence and pelvic/loin pain with or without visible cervical lesion on speculum examination. HIV infected person was defined as a person who was HIV seropositive as per recommended Tanzanian National AIDS Control Programme HIV diagnostic testing algorithm [15]. Tumor cell differentiation was defined as the extent to which the cancer cells resembled comparable normal cells, and was categorized into well-differentiated, moderately differentiated and poorly differentiated tumours.

Data were collected using pre-tested structured questionnaires. Information collected through participants interview included demographic characteristics (age, marital status, parity, educational level, occupation, region of residence), parity of the participant, age at first pregnancy, age at sexual debut, use of modern contraceptives and smoking. In addition, blood was taken from each participant for laboratory investigations: complete blood count, serum Creatinine level, and HIV antibody test. Examination under anaesthesia for disease staging was done as well as taking a punch biopsy from cervical lesion for histological examination. Biopsy was preserved in 10% formalin. Histological examination was done by light microscopy under Haematoxylin and Eosin stain.

Pre and post-test counseling and examination for HIV followed the available national guidelines [15]. SD-Bioline (Standard Diagnostics, Inc. Kyonggi-do, Korea) was used for testing HIV among patient with cancer of the cervix and positive results were further confirmed by Determine HIV 1&2 serum/plasma assays (Abbott Diagnostic Division, Tokyo Japan). Undetermined results were further tested using Uni-Gold Recombigen™ HIV rapid test as per existing nation algorithm for HIV testing. CD4+ count estimation was done using CD4+ analyzer-Facs counts (BD Biosystem USA) as per manufacturer’s protocol. The test is routinely done to all newly diagnosed HIV positive patients to determine their eligibility into treatment. Chest x-rays and abdominal/pelvic ultrasound were taken for the purpose of staging.

Collected data were entered into EpiData version 3.1, and then exported into STATA version 11 for cleaning, consistency checks and analysis. A t-test statistic was used to analyze continuous data into means, standard deviations and confidence intervals. Categorical variables were summarized into proportions and compared using Chi-square statistical test and Odds ratios. Binary logistic regression was done to determine risk factors for cervical cancer. Factors with p-value less than or equal to 0.05 were retained for multivariate logistic regression to calculate their odd ratios and confidence intervals. Respective p-values at 95% confidence level are also reported.

Ethical review and approval was sought and obtained from Joint Weill Bugando University and Bugando Hospital ethical, research and publication committee. Informed consent was requested and obtained from all participants in Kiswahili language. HIV-positive women were referred to the HIV/AIDS care and treatment unit at the hospital for further management.

## Results

A total number of 106 patients with a suspected clinical diagnosis of cervical cancer were seen in the Gynecological ward/clinic at Bugando Medical Centre during the study period. Of these, 15 patients were excluded from the study (8 patients did not consent for HIV testing and 7 patients had communication problems). Therefore a total of 91 patients were recruited in the study.

Of the 91 patients with suspicious cervical lesions, 74 (81.3%) had histological confirmation of cervical cancer, and thus included in the analysis. The mean age of participants with cervical cancer was 50.5 ± 12.5 years (range 29–85 years). The level of education among study participants was low, with almost half (52.7%) of all participants having no formal education and just 2(2.7%) educated beyond primary education. The majority 58 (78.3%) were peasant farmers (Table 1).

**Table 1:** Socio-demographic and Reproductive characteristics of the study population

**Characteristics**	**HIV + (n = 22)**		**HIV- (n = 52**)		**Total (n = 74)**	
	**Number**	**Mean ± SD**	**Number**	**Mean ± SD**	**Number**	**Mean ± SD**
Age in years		40.2 ± 8.6		54.9 ± 11.4		50.5 ± 12.5
Age group						
≤35	7 (31.8)		1 (1.9)		8 (10.8)	
36-45	10 (45.5)		9 (17.3)		19 (25.7)	
46-55	3 (13.6)		18 (34.6)		21 (28.4)	
56+	2 (9.1)		24 (46.2)		26 (35.1)	
Education level						
No formal education	5 (22.7)		34 (65.4)		39 (52.7)	
Primary education	16 (72.7)		17 (32.7)		33 (44.6)	
Secondary education	1 (4.5)		1 (1.9)		2 (2.7)	
Marital status						
Married	11 (50.0)		32 (61.5)		43 (58.1)	
Widow/Separated	11 (50.0)		20 (38.5)		31 (41.9)	
Occupation						
Peasant/farmer	13 (59.1)		45 (86.5)		58 (78.3)	
Housewife	3 (13.6)		0 (0)		3 (4.1)	
Professional salaried job	1 (4.5)		2 (3.8)		3 (4.1)	
Petty trader	5 (22.7)		5 (9.6)		10 (13.5)	
Area of residence						
Mwanza	13 (59.1)		23 (44.2)		36 (48.6	
Mara	4 (18.2)		11 (21.2)		15 (20.3)	
Shinyanga	4 (18.2)		11 (21.2)		15 (20.3)	
Others	1 (4.5)		7 (13.5)		8 (10.8)	
Age at Marriage						
<18 years	10 (45.5)		31 (59.6)		41 (54.4)	
≥18 years	12 (54.5)		21 (40.4)		33 (45.6)	
Parity						
≤1	2 (9.1)		1 (1.9)		3 (4.1)	
2-5	2 (9.1)		13 (25.0)		20 (27.0)	
>5	18 (81.8)		38 (73.1)		51 (68.9)	
History of STIs						
Yes	8 (36.4)		7 (13.5)		15 (20.3)	
No	14 (63.6)		45 (86.5)		59 (79.7)	
Lifetime sexual partners						
1	2 (9.1)		18 (34.6)		20 (27.0)	
>1	20 (90.9)		34 (65.4)		54 (73.9)	
Contraceptive use						
None	8 (36.4)		41 (78.8)		49 (66.2)	
Pills	9 (40.9)		7 (13.5)		16 (21.6)	
Barrier	0 (0)		1 (1.9)		1 (1.4)	
Implants	3 (13.6)		2 (3.8)		5 (6.8)	
Depo provera	2 (9.1)		1 (1.9)		3 (4.0)	

Note: Number in brackets denotes respective percentages.

Of all cervical cancer patients, 44 (56.4%) were already married by the age of 18 years and 51(68.9%) of participants had delivered more than five children in their lifetime. The median parity was 7.0 (not shown in Table 1). History of a sexual transmitted infection was reported in 15 (20.3%) participants. Ever use of hormonal contraception was reported in 25 (33.8%) of the study participants, although it was not possible to accurately record the duration of use (Table 1).

Of the total 74 cervical cancer confirmed cases, 39 patients (52.7%) were in early stages of the disease: 24(32.4%) in stage IB and 15 (20.3%) in stage IIA). Of the remaining 35 patients with late stage disease, 11 (14.9%) were in stage IIB, six (8.1%) in stage IIIA, ten patients (13.5%) in stage IIIB and eight patients (10.8%) in stage IVA (Table 2). Abnormal vaginal discharge and contact vaginal bleeding were the most common clinical presentations among participants reported by 63 (85.1%) and 61 (82.4% patients respectively. Thirty six percent of the participants had postmenopausal bleeding; approximately 43 (58.1%) had ulcerative types of cervical lesions and 34 (45.9%) had fungating lesions (not shown in tables).

**Table 2:** HIV seropositivity in relation to some selected participant characteristics (N = 74)

**Characteristic**	**HIV serostatus**		**Total**	**p-value**
	**Positive**	**Negative**		
Age group				
≤50 years	19 (50.0)	19 (50.0)	38 (100.0)	<0.001 (Pearson)*
>50 years	3 (8.3)	33 (91.7)	36 (100.0)	
Sub-total	22 (29.7)	52 (70.3)	74 (100.0)	
Clinical stage				
Stage IB	5 (20.8)	19 (79.2)	24 (100.0)	
Stage IIA	4 (26.7)	11 (73.3)	15 (100.0)	
Stage IIB	4 (36.4)	7 (63.6)	11 (100.0)	
Stage IIIA	3 (50.0)	3 (50.0)	6 (100.0)	
Stage IIIB	4 (40.0)	6 (60.0)	10 (100.0)	
Stage IVA	2 (25.0)	6 (75.0)	8 (100.0)	
Total	22 (29.7)	52 (70.3)	74 (100.0)	
Stage classification				
Early stage (IA-IIA)	9 (23.1)	30 (76.6)	39 (100.0)	0.186 (Pearson)**
Advanced stage (IIB-IVB)	13 (37.1)	22 (62.9)	35 (100.0)	
Total	22 (29.7)	52 (70.3)	74 (100.0)	
Tumor cell differentiation				
Well	12 (52.2)	11 (47.8)	23 (100.0)	0.006 (exact)***
Moderate	8 (25.8)	23 (74.4)	31 (100.0)	
Poor	2 (11.1)	16 (88.9)	18 (100.0)	
Total	22 (30.6)	50 (69.4)	72 (100.0)****	

Note: Number in brackets denotes percentages.

*p-value comparing HIV seropositivity between participants aged ≤ 50 years versus those aged over.

**p-value comparing HIV seropositivity between women with early versus late stage disease.

***p-value comparing participants with well differentiated and combined moderately and poorly differentiated tumor cells.

****Two patients had small cell carcinoma whose cellular differentiation could not be determined.

Squamous cell carcinoma was the commonest type of cancer comprising of 69 (93.2%) of the total 74 participants. Other histological types of cervical cancer were 3 (4.1%) adenocarcinoma and 2 (2.7%) small cell carcinoma. Overall, HIV seropositivity among participants with cervical cancer was 29.7%. The level of HIV seroprevalence in the various diseases stages were 22.7% (5 of the 24 participants in stage 1b), 26.7% (four of the fifteen participants in stage IIa), 36.4% (four of the eleven participants in stage IIb) and 50.5% (three of the six in stage IIIa); 40.0% (four of the ten patients in stage IIIb and two of the total eight patients in stage IVa (25.0%).

Thirty six patients were aged over 50 years versus thirty eight patients who were aged 50 years and less. Three of the thirty six patients (8.3%) aged over 50 years were HIV positive compared to nineteen of the thirty eight patients (50.0%) aged 50 years and less (p < 0.001, Table 2). There were 23 patients with well differentiated tumor cells (31.9%) compared to 49 patients with moderately and poorly differentiated cancer cells. Of the total 23 patients 12 (52.2%) were HIV + compared to ten patients among the 49 patients (20.4%) with moderately and poorly differentiated tumor cells and the difference of seropositivity was statistically significant (p = 0.006).

The mean CD4+ count of the 22 HIV positive participants was 315.0 ± 138.0. Overall, 68.2% of all participants had CD4+ counts of ≤ 350. On average, HIV + patients with early stage disease (stage IA-IIA) had more CD4+ count than those with advanced disease (Stage IIB-IVB) (mean 385.8 ± 170.4; 95% CI 354.8-516.7 and 266.2 ± 87.5; 95% CI 213.3-319.0 respectively, p = 0.042) (Table 3). In contrast, the average CD4+ count of the 12 patients with well differentiated tumor cells did not differ significantly from the combined ten patients with moderately and poorly differentiated tumor cells (mean CD4+ count 318 ± 148.0, 95% CI 224.8-412.9 versus 310.6 ± 132.8, 95%CI 215.6-405.6; p = 0.893 respectively).

**Table 3:** CD4+ distribution, level of differentiation and Cancer stage among HIV + participants (n = 22)

**Tumor characteristic**	**CD4+ count**		**Total**	**Mean CD4+ count ± SD**	**95% CI**	**p-value**
	**≤350**	**>350**				
Cellular differentiation						
Well	8 (66.7)	4 (33.3)	12 (100.0)			
Moderate	5 (62.5)	3 (37.5)	8 (100.0)			
Poor	2 (100.0)	0 (0)	2 (100.0)			
Total	15 (68.2)	7 (31.8)	22 (100.0)			
Overall CD4 count n = 22				315.0 ± 138.0		
Well differentiated n = 12				318.8 ± 148.0		
Moderately and poorly differentiated n = 10				310 ± 132.8		0.893*
Cancer stage						
Early stage ( IA-IIA)	5 (55.6)	4 (44.4)	9 (100.0)	385.8 ± 170.4	354.8-516.7	0.042**
Late stage (IIB-IVB)	10 (76.9)	3 (23.1)	13 (100.0)	266.2 ± 87.5	213.3-319.0	
Total	15 (68.2)	7 (31.8)	22 (100.0)			0.290 (Pearson)***

Note: Number in brackets denotes%.

* p-value from a t-test statistic comparing the mean CD4+ count between well differentiated and

moderate and poorly differentiated tumor cell patients.

** p-value from a t-test statistic comparing the mean CD4+ count between early and late stage disease.

***p-value comparing women with CD4+ count ≤350/μL in early and late stage diseases.

Factors associated with HIV seropositivity were explored in a binary logistic regression analysis (Table 4). History of STI, ever use of modern contraception, aged over 50 years and previous history of multiple sexual partners were significantly associated with HIV seropositivity. Except for a strong evidence to suggest that ever use of modern contraception was associated with cancer cell differentiation (comparing poorly differentiated tumor cells and combined well and moderately differentiated cells patients), there was no evidence to suggest an association between cancer cell differentiation and history of STI, age of more than 50 years or history of lifetime multiple sexual partners (Table 4).

**Table 4:** Logistic regression model for factors associated with poor cell differentiation and HIV seropositivity

**Factor**	**OR**	**95% CI**	**p-value**
1: binary analysis			
Factors associated with cell differentiation			
Previous history of STI versus none	1.39	0.39-4.90	0.623
History of modern contraceptive use versus never used	0.16	0.06-0.49	0.001
Age at first delivery before 18 years versus ≥ 18 years	0.64	0.23-1.78	0.389
History of multiple sexual partners in one’s lifetime versus none	0.98	0.32-3.01	0.968
Age > 50 years versus ≤ 50 years	1.91	0.70-5.24	0.209
Factors associated with HIV serostatus			
Previous history of STI versus none	3.43	1.09-10.79	0.035
History of modern contraceptive use versus never used	5.79	1.99-16.83	0.001
Age at first delivery before 18 years versus ≥18 years	1.78	0.64-4.91	0.272
History of multiple sexual partners in one’s lifetime versus none	5.56	1.18-26.25	0.030
Age > 50 years versus ≤50 years	0.09	0.02-0.36	0.001
2: HIV and cancer cellular differentiation n = 72			
Binary (unadjusted) logistic regression: HIV and cancer cell differentiation (poorly differentiated versus well and moderately differentiated)	0.21	0.04-1.02	0.053
Multivariate (adjusted) logistic regression: HIV and cancer cell differentiation (poorly differentiated versus well and moderately differentiated) *	5.62	1.76-17.94	0.004

* adjusted for ever used modern contraception or not.

In unadjusted binary logistic regression model comparing poorly differentiated tumor cell patients versus combined well and moderately differentiated tumor cell patients, there was weak evidence to suggest an association between HIV seropositivity and cancer cell differentiation (OR 0.21 95% CI 0.04-1.02, p = 0.053). Ever use of hormonal contraception- a factor associated with both HIV and cancer cell differentiation was adjusted for in a multivariate logistic regression model. There was strong evidence to suggest that HIV seropositivity was associated with cancer cellular differentiation after the adjustment (OR 5.62 95%CI 1.76-17.94, p = 0.004) (Table 4), indicating an almost six fold likelihood of HIV seropositivity among women with poorly differentiated tumor cells compared to those with well and moderately differentiated cells.

## Discussion

Results from this study suggest that women with poorly differentiated cancer cells are more likely to be HIV positive than those with well and moderately differentiated cell types, especially after adjusting for ever use of hormonal contraception. The finding of almost a six times more likelihood of HIV infection among these women suggests an additional risk besides the known poorer prognosis [16]. An association of poorly differentiated cervical tumor cells and HIV has been reported in a study done in Kenya [16].

In this study; HIV seropositivity among participants with cervical cancer was 29.7%. The majority of the participants with cervical cancer were in the age groups less than 50 years, and the finding is similar to reports from other studies [5,9]. The high prevalence of cervical cancer in participants aged less than 50 years in this study may be influenced by the high HIV seropositivity in this group, as HIV has been shown to cause rapid progression of cervical cancer [8-11]. Nevertheless, the nature of the study does not allow causal inferences.

Most of the HIV positive patients (68.2%) had a CD4+ count of less than 350cells/μl indicating the need for HIV screening of all cervical cancer patients so that appropriate treatment is initiated concurrent with cervical cancer management. CD4+ count of 350/μL and less was seen more often among women with advanced cervical cancer than those who had more counts although the difference was not statistically significant. This is contrary to other study findings in which a low CD4+ count was associated with advanced cervical cancer stages at presentation [8,10,11].The difference may be attributed to the small sample size in our study.

The histopathology findings of squamous cell carcinoma as the most common type of cervical cancer in our study is similar to studies done in Kenya, Uganda and South Africa in which as high as 80% of cases had squamous cell carcinoma [13,16-18]. In contrast, the finding of non-squamous cervical cancer in this study of just 8.7% is lower than the 11% reported in Nigeria [19].

The limitations of this study are worth mentioning. The sample size of this study was small and the nature of the study does not allow causal inferences. Participants were women who sought care at a health unit during the study period. Although we believe that their characteristics, including the outcomes of interest may not differ from the population of women with similar conditions who seek care at the hospital annually, the findings may not be extrapolated to the pattern of the disease countrywide.

## Conclusion

Results from this study suggest that HIV seropositivity is associated with cervical cancer cell differentiation and that HIV co-infected women present with relatively low CD4+ count. More studies are needed in this and similar settings to reliably document this relationship so that clear guidelines for the management of cervical cancer patients co-infected with HIV are developed. In the interim, HIV screening should be encouraged among all cervical cancer patients to enable timely linkage to appropriate care, treatment and support.

## Ethical approval

Ethical review and approval was sought and obtained from a Joint Weill Bugando University and Bugando Medical Center Research and Publication Committee.

## Consent

All participants were provided with consent information sheet and forms to read and consent for participation, but for non literate women, the consent sheet was read aloud in Kiswahili by the recruiter who was not part of the health care provision team to the woman. After agreeing to participate, her thumbprint was stamped on the consent form to signify her consent.

## Competing interests

The authors declare that they have no competing interests.

## Authors’ contributions

DM: Main author of the study, involved in design, writing the proposal, data collection, analysis and preparation of the manuscript. PR: Involved in development of proposal, data analysis together with main author in review of histological slides and manuscript preparation. AM: Involved in developing the proposal, data collection, clinical staging and preparation of the manuscript. MM: Involved in developing the proposal, data collection, analysis and preparation of the manuscript. NM: Involved in preparation of the study, data collection and analysis. All the authors read and approved the final manuscript.
